# ‘It’s not making a decision, it’s prompting the discussions’: a qualitative study exploring stakeholders’ views on the acceptability and feasibility of advance research planning (CONSULT-ADVANCE)

**DOI:** 10.1186/s12910-024-01081-5

**Published:** 2024-07-23

**Authors:** Victoria Shepherd, Kerenza Hood, Fiona Wood

**Affiliations:** 1 Centre for Trials Research, Cardiff University, Cardiff, UK; 2 PRIME Centre Wales, Cardiff, UK; 3 Division of Population Medicine, Cardiff University, Cardiff, UK

**Keywords:** Advance planning, Decisional capacity, Research, Informed consent, Under-served populations, Qualitative

## Abstract

**Background:**

Health and care research involving people who lack capacity to consent requires an alternative decision maker to decide whether they participate or not based on their ‘presumed will’. However, this is often unknown. Advance research planning (ARP) is a process for people who anticipate periods of impaired capacity to prospectively express their preferences about research participation and identify who they wish to be involved in future decisions. This may help to extend individuals’ autonomy by ensuring that proxy decisions are based on their actual wishes. This qualitative study aimed to explore stakeholders’ views about the acceptability and feasibility of ARP and identify barriers and facilitators to its implementation in the UK.

**Methods:**

We conducted semi-structured interviews with 27 researchers, practitioners, and members of the public who had participated in a preceding survey. Interviews were conducted remotely between April and November 2023. Data were analysed thematically.

**Results:**

Participants were supportive of the concept of ARP, with differing amounts of support for the range of possible ARP activities depending on the context. Six main themes were identified: (1) Planting a seed – creating opportunities to initiate/engage with ARP; (2) A missing part of the puzzle – how preferences expressed through ARP could help inform decisions; (3) Finding the sweet spot – optimising the timing of ARP; (4) More than a piece of paper – finding the best mode for recording preferences; (5) Keeping the door open to future opportunities – minimising the risk of unintended consequences; and (6) Navigating with a compass – principles underpinning ARP to ensure safeguarding and help address inequalities. Participants also identified a number of implementation challenges, and proposed facilitative strategies that might overcome them which included embedding advance research planning in existing future planning processes and research-focused activities.

**Conclusions:**

This study provides a routemap to implementing ARP in the UK to enable people anticipating impaired capacity to express their preferences about research, thus ensuring greater opportunities for inclusion of this under-served group, and addressing the decisional burden experienced by some family members acting as proxies. Development of interventions and guidance to support ARP is needed, with a focus on ensuring accessibility.

**Supplementary Information:**

The online version contains supplementary material available at 10.1186/s12910-024-01081-5.

## Background

There is an increasing focus on supporting people to discuss and express their preferences about their future care and treatment prior to any loss of capacity. This can help provide clarity about what care and treatment the person should be provided with when they lose the ability to communicate [1]. This may be of particular importance for people living with conditions that may affect future decisional capacity such as dementia, people receiving palliative or end-of-life care, or people who more generally wish to plan ahead in anticipation of any ageing-related changes to cognition. With a global ageing population, the number of people aged 65 + years is projected to double to 1.5 billion by 2050 [2] and is likely to be accompanied by an increase in the prevalence of disease and disability, including cognitive impairment [3]. This has led to even greater attention being paid to how we can ensure that actions and decisions made on behalf of a person who lacks capacity are in accordance with their previously expressed wishes and preferences [4].

### Advance planning for health and care

For future treatment and care decisions, advance care planning (ACP) is accepted as a useful anticipation tool [5]. It enables individuals to define their goals and preferences for their future medical treatment and care, to discuss these goals and preferences with family and healthcare providers, and to record and review these preferences if appropriate [6]. The National Institute for Health and Care Excellence (NICE) recommends ACP in its guidelines for decision-making and mental capacity (NG108) [6], and ACP is embedded across NICE guidance for end-of-life care for adults, dementia, and mental health conditions such as schizophrenia [7].

In England and Wales, ACP activities can also include people creating a legally binding ‘Advance Decision to Refuse Treatment’ (ADRT) to guide their care (Mental Capacity Act 2005 (MCA) s24-26 [8], or expressing their non-binding preferences through an advance statement which can guide future care decisions [9]. People are also able to nominate a Lasting Power of Attorney (LPA) for health and welfare which involves giving one or more people legal authority (acting jointly or severally) to make decisions on their behalf if they are unable to do so, including consent to care and treatment decisions (MCA s9) [8]. Decisions made under the authority of the LPA must be in the person’s best interest, and an attorney acting under the authority of an LPA must consider the person’s past and present wishes and feelings, beliefs, and values (MCA s4) [8]. Although arrangements differ across jurisdictions in the UK.

The same legal frameworks that govern health and care for people lacking capacity require that decisions about research participation should also be based on their wishes and preferences [8]. This includes the requirement that researchers must respect any advance decisions and expressed preferences, wishes, or objections that the person has made in the past (MCA Code of Practice s 11.44) [10]. However, currently in the UK and many other countries, ACP discussions and LPA arrangements do not currently extend to decisions about research participation.

### Advance planning for research

Advance research planning (ARP) is an umbrella term for a process in which people anticipating loss of capacity are encouraged to think about, discuss and document their preferences for taking part in research in the future [11]. As a broad spectrum of activities, it may include making an advance research directive and naming a trusted person who they wish to be involved in decisions about research participation [11]. The person may express their general views about taking part in research in the future or, under some versions of ARP, their wish about being involved in a specific research project should they have lost capacity to consent at the point they become eligible (alternatively described as ‘advance consent’) [12].

ARP may have a number of benefits. It may promote and extend personal autonomy - if it is deemed important for decisions made on behalf of a person to be based on their wishes and preferences this should also include decisions about participation in research [13]. Participating in research is often perceived as being beneficial for the person themselves, such as gaining access to new treatments or giving a sense of hope, as well as contributing to the wider societal benefits, with altruism often being a motivating factor for people to participate [14]. ARP may extend their ability to contribute to research in the event that they are unable to communicate their decision, or conversely to ensure that people are not included in research that they would not wish to participate in.

ARP may also address concerns that alternative decision makers (usually a family member acting as a consultee [15] or legal representative [16]) are inclined to make decisions about research that are not in accordance with the person’s wishes – either enrolling them in a study they would not wish to participate in or declining a study they would consent to if able [17, 18]. It may also improve the confidence of alternative decision makers and therefore reduce the emotional and decisional harms they often experience when making difficult decisions about research participation, or the burden they may perceive [19–21]. It may also support inclusion in emergency research where it is not possible to involve an alternative decision maker due to the time-critical nature of the intervention [12]. This in turn could also address the frequent exclusion of adults with impaired capacity to consent from research which is often due to these ethical and legal uncertainties [22], and alternative decision makers’ underestimation of people’s willingness to be included in research should they lack capacity [17] which has consistently been shown to be high [17, 23, 24]. Although people’s willingness to be included varies considerably depending on the type of research, the activities involved, and the likely risks and benefits of participation [24].

ARP also raises a number of questions, including around how likely it is that people are able to imagine what it would be like to participate in research with significant cognitive impairment - it has previously been described as a ‘rare person’ who could actually achieve this level of understanding [13] – and how these wishes could be interpreted by an alternative decision maker [25]. There are also ethical concerns about whether the informational standard required for any prior decision contained in an advance research directive could be sufficiently informed to constitute consent, which raises questions about the validity of any choices made about participation [26] and the risk of being included in research that they would not agree to (or no longer would agree to). Introducing advance research planning into existing processes also raises concerns around the risk of exacerbating therapeutic misconception and introducing additional legal complexities [25].

There are also a number of implementation questions. Despite advance care planning being embedded in national guidance and having a long history, it is not widely taken up, even amongst older patients and those who have recently been in hospital [27]. This is due to a lack of knowledge and awareness about ACP, and when coupled with misunderstandings and mistrust in the system may particularly impact uptake amongst diverse communities [28]. Advance planning for research is a far less familiar concept, and as such it has yet to be fully implemented - even in countries where much rigorous preparatory work has been undertaken with a range of stakeholders, such as in Australia [11, 24, 29, 30].

### Need for advance research planning in the UK

Recommendations for introducing ARP in the UK were first made in 2009 when the Nuffield Council on Bioethics called for advance planning arrangements to be amended to enable people to make a (non-binding) advance statement about research participation, and for the role of the health and welfare attorney in England and Wales be explicitly extended to include decisions about research [31]. More recent recommendations from researchers investigating end-of-life care research suggested that patients should have opportunities to discuss and document their preferences and wishes about research participation, and those who are likely to lose capacity should be asked to designate a consultee to provide an opinion on their participation in a study [32]. ARP has already gained some ethical and legal recognition internationally, including in Australia, USA, Canada, and beyond [11], although there are notable gaps between legislation and policy and practice [25]. As the UK Government has, through the National Institute for Health and Care Research (NIHR), a strategic aim for research to be integrated into care pathways within the NHS [33], and there is an international focus on creating ‘learning health systems’ to enable continuous improvement in health care [34], it is an opportune time to explore opportunities for people anticipating future incapacity to be able to express their wishes and preferences about future research as well as their treatment and care.

Following on from a recent survey exploring public and professional stakeholders’ views about the acceptability and feasibility of advance research planning in the UK which found high levels of support [23], we conducted a qualitative study to explore in more depth the introduction of ARP in the UK and issues surrounding its implementation.

## Methods

### Aim

This study aimed to develop an understanding of stakeholders’ views about the acceptability and feasibility of advance research planning and identify barriers and facilitators to the implementation of advance research planning in the UK.

### Design

The CONSULT-ADVANCE study forms part of a larger programme of research exploring the ethical, legal, and methodological issues surrounding research involving adults with impaired capacity to consent (CONSULT) [35]. The study consisted of two phases - a cross-sectional online survey followed by semi-structured interviews which were informed by the survey findings. Both phases were conducted with a range of stakeholders including members of the public (including those with personal experience of conditions that may affect capacity to consent), researchers, and other professionals with an interest in research into capacity-affecting conditions. The methods and findings from the survey phase have previously been reported in full [23]. This paper reports the interview phase which followed on from the survey and enabled a more in-depth exploration of the issues identified in the survey. The research questions for this phase of the study were: what are public and professional stakeholders’ views about the introduction of advance research planning in the UK, and what barriers and facilitators might be encountered?

### Sampling

Participants for the interview phase were identified through the online survey in which participants were able to express their interest in being contacted to take part in an interview. Survey participants were recruited through a variety of routes, including social media platforms (Twitter/X), charitable organisations (Parkinson’s UK, Stroke Association), research registries (Join Dementia Research), research networks (MRC-NIHR Trials Methodology Research Partnership, British Society for Gerontology, Dementia Researcher), and invited through organisations such as the Health Research Authority (HRA).

As the purpose of the interviews was to obtain a range of personal and professional views and experiences, interview participants were sampled purposively from those who participated in the survey using maximum variation sampling to include a range of perspectives [36]. Participants were iteratively selected to take part in an interview based on criteria used to construct the sample frame such as their role (e.g. people living with a capacity-affecting condition, family member, member of the public, health care professional (HCP or researcher), experience of capacity-affecting conditions or disabilities (e.g. dementia, learning disability, stroke), and demographic characteristics with a particular focus on ensuring a diverse range of perspectives were included (e.g. age, gender, ethnicity, geographical location).

### Recruitment

Potential participants were contacted by email and provided with the Participant Information Sheet and a copy of the consent form and offered the opportunity to ask questions or to have a preliminary meeting with the lead researcher if they would like to discuss the study in the first instance. If they were willing to participate in an interview this was arranged at a convenient time and could be by telephone or via a secure video conferencing platform (e.g. Zoom) depending on participant preference.

### Ethical considerations

Ethical approval was obtained from the School of Medicine Research Ethics Committee at Cardiff University (SMREC ref. 22.84) prior to commencing the study. As interviews were being conducted remotely, informed consent was obtained verbally prior to the start of the interview, and the process audio recorded. The statements on the consent form were read out by the researcher to the participant who confirmed that they agreed to each statement. This corresponds with guidance from the HRA that consent may be obtained orally (or by any other means of communication) for research studies that are not clinical trials [37].

### Data collection

A topic guide (Supplementary File 1) was developed to help structure the interview questions. It was informally piloted with a small number of people (*n* = 4) drawn from similar groups to the participants to check the ordering and wording of the questions. No changes were made to the topic guide following this piloting. Interview questions explored participants’ views on ARP in general, what activities ARP should include, who should be involved, and how and when it might best be undertaken. Participants were also encouraged to talk about other areas and challenges that they felt were relevant to their experiences. Their views were also sought on what information those involved might need in order to effectively engage in ARP and in what format, and the best routes for implementation and for raising awareness.

At the start of the interview, participants were provided with a reminder about the concept of ARP being used in the study, which was broadly described as being ways to help people to express what their wishes about future research participation are should they be unable to make their own decision, and who they would like to make a decision about research on their behalf.

Data collection took place between April 2023 and October 2023. Interviews were conducted by the lead author, audio recorded, and transcribed verbatim by an external transcription service.

### Data analysis

Data generation and analysis were undertaken concurrently using an iterative approach to coding and identification of preliminary themes. Participants were allocated a unique study identifier and any identifying features were removed from transcripts prior to analysis to ensure anonymity. The transcripts were checked for accuracy and completeness against the source data.

Thematic analysis was conducted through a process of identifying, analysing, and reporting themes [38]. Following familiarisation with the data, the transcripts were iteratively coded by the lead author using an inductive coding approach supported by the use of a qualitative data analysis tool (NVivo 12, QRS International) to manage the data. Developments in the analytical process were recorded through data analysis memos held in NVivo.

Preliminary analysis of the first four interview transcripts was undertaken and discussed with the wider research team to review and refine the coding framework and coding process, and this was reviewed again after the first 20 interviews had been conducted. Recruitment and data collection continued until the research team considered that sufficient data (defined as the depth, diversity, and adequacy of the data) had been collected in order to answer the research question(s) [39]. This was considered to have been reached after 27 interviews, as determined through agreement between the study team. The themes were then reviewed and finalised.

## Results

Twenty-seven participants were interviewed. Interviews were predominantly conducted via video using Zoom (*n* = 24) with a mean duration of 38 min (range 26–53 min). Participant characteristics are shown in Table 1 below.

**Table 1:** Characteristics of interview participants

Participant characteristic	*n* = 27 (%)
**Gender**	
Female	21 (78)
Male	6 (22)
Prefer to self-describe	0
**Age**	
18–24	1 (4)
25–34	5 (19)
35–49	6 (22)
50–64	10 (37)
65+	5 (19)
**Geographical location**	
England	17 (63)
Wales	8 (30)
Scotland	1 (4)
Northern Ireland	1 (4)
**Ethnicity**	
White	23 (85)
Asian / Asian British	1 (4)
Black / African / Caribbean / Black British	1 (4)
Mixed / Multiple ethnic groups	0
Other ethnic group (self-described)	2 (7)
**Stakeholder group (primary)**	
Researcher	7 (26)
Health care professional	4 (15)
Family member/friend of someone with an impairing condition	6 (22)
Member of the public	6 (22)
Experience of living with a condition affecting memory/understanding	2 (7)
Other	2 (7)
**Area of interest**^	
Dementia	15 (56)
Parkinson’s disease	4 (15)
Stroke	5 (19)
Palliative or end of life care	5 (19)
Care of older people	2 (7)
Care homes	2 (7)
Other (e.g. Persistent Disorders of Consciousness, intellectual disability, emergency care, Huntington’s disease, REC)	5 (19)

^Participants could select more than one option

Participants were very supportive of advance research planning (ARP) as a concept, whilst recognising that it may take various different forms or include a range of activities in practice. Views about the feasibility of ARP varied depending on these different contexts. Six main themes were identified which describe their views about ARP, the purpose of ARP and the processes involved, and the barriers and enablers to its implementation: (1) ***Planting a seed*** – creating opportunities to initiate or engage with ARP; (2) ***A missing part of the puzzle*** – how preferences expressed through ARP could help inform decisions about participation; (3) ***Finding the sweet spot*** – optimising the timing of ARP; (4) ***More than a piece of paper*** – finding the best mode for recording and documenting preferences; (5) ***Keeping the door open to future opportunities*** – minimising the risk of unintended consequences; (6) ***Navigating with a compass*** – principles underpinning ARP to ensure safeguarding and help address inequalities. A number of sub-themes were also identified.

Participants also recognised the complexities and implementation challenges that might be encountered in real world settings, and proposed a number of facilitative strategies that might help address them.

### Theme 1: planting a seed – creating opportunities to initiate or engage with ARP

#### ARP as a promising contribution to research

Whilst ARP was a new concept to almost all participants, there were strong levels of interest in enabling people to express their preferences about future research participation. ARP was viewed as having a valuable role in both supporting advance planning arrangements and maintaining opportunities to be involved in research.

*‘I am working in the palliative and end of life research world*,* and I was struck by how brilliant a concept that was. We are making great strides in trying to advance people’s forward planning when it comes to their own death. So*,* things like research and the ability to be useful and have some kind of utility and impact*,* either whilst you’re dying or incapacitated or after your death. Certainly*,* from a personal point of view I would love to have something like that in place so that I can continue to be useful in some way’ [ID 06*,* researcher]*.

Participants reported examples of ARP that had already occurred in practice. This included people who had sought opportunities to express their continued willingness to take part in future research such as including a statement in their LPA. It also included researchers who had introduced conversations about future research participation with patients who were seriously ill during the COVID-19 pandemic, and some palliative and end of life care studies that had included statements about continued participation in the event of incapacity in the consent process.

*‘I eventually got round to . [completing my own]… Lasting Power of Attorney*,* and under the statement about “Is there any specific wishes?” the only wish that I have put is that if there is a research opportunity then I want the opportunity [to be considered]. If the doctors think it’s in my best interests or they are happy that I would be a participant*,* then just do it’ [ID 09*,* member of the public]*.

*‘Down on the wards [during COVID-19 pandemic] we were asking people*,* “If you were to deteriorate*,* become unwell*,* and the doctors needed to put you to sleep with a tube to help you breathe*,* would you reconsider research at that point?” and we started documenting that in patients notes*,* so there was some kind of guide for the ICU team to go off. I don’t know how helpful it was or how much they were looking at it*,* but it felt like we were doing something directly to try and protect people’s kind of rights and what they wanted’ [ID 01*,* HCP]*.

Participants described how conversations about future preferences about care and treatment were often avoided in society due to cultural attitudes towards serious illness and death. Participants viewed conversations about research preferences as also being important, but voiced the view that they were even less likely to occur than conversations about other aspects of future planning such as options for medical treatment. Some researchers reported seeing the impact of this lack of prior discussion in practice, describing how family members can find themselves in a situation where they are unable to draw on a previous discussion to help them to make a decision about research participation.

*‘It’s again a huge part of our problem that we don’t have these conversations about end-of-life care*,* emergency care*,* what happens if I lack capacity …. and then when you do ask a consultee*,* they’re like ‘well we never talked about it*,* we never had this conversation’ …. certainly no one’s having chats about research*,* your general public are not having these conversations’ [ID 11*,* researcher]*.

### Opportunities to initiate ARP discussions

Rather than ARP being viewed as a one-off opportunity or a single event, participants thought it was important to consider how to maximise opportunities for people to have conversations about their research preferences. Having opportunities to start initial conversations about future research was seen as key, although participants recognised that it could be a potentially distressing topic for people to discuss, particularly if it was in the context of having been diagnosed with a condition that may affect their decision-making ability in the future.

*‘The vast majority of people haven’t really even thought about it. So*,* it just plants a seed there*,* and the seed may well come to fruition; it may not. Hopefully it wouldn’t*,* because I wouldn’t get anything that needs studying*,* but it’s just there*,* it gets the discussion going’ [ID 14*,* family member]*.

Opportunities for ARP could take the form of an introductory discussion with the person and their family members, led by a member of their clinical care team such as a GP or another practitioner. This might lead to more formal processes such as creating a documented record of the discussion and the person’s preferences should those involved wish to do so.

*‘I think pragmatically*,* for me*,* it would be more about having a discussion with family*,* and … obviously people can write it down if they wish’ [ID 05*,* researcher]*.

Additional opportunities to have discussions about ARP might arise when people are engaging in other future planning activities (e.g wills, funeral preferences, Lasting Power of Attorney arrangements, organ donation), research related activities (e.g taking part in a specific study, signing up to a research registry or a biobank) or other altruistic voluntary acts (e.g donating blood), as well as being embedded in communications that come from charities and other organisations (e.g condition-specific organisations such as Alzheimer’s Society, peer support groups, or community initiatives such as Park Run).

*‘If you give people as many opportunities to engage with the process the better …. because most people don’t want to think about advanced things to do with their death and dying until they’re right up there confronted with it’ [ID 06*,* researcher]*.

*‘There is a place for it within peer groups*,* within third sector groups*,* within charities and national foundations to sort of signpost people to the idea of doing it’ [ID 12*,* HCP]*.

**Fig. 1:**
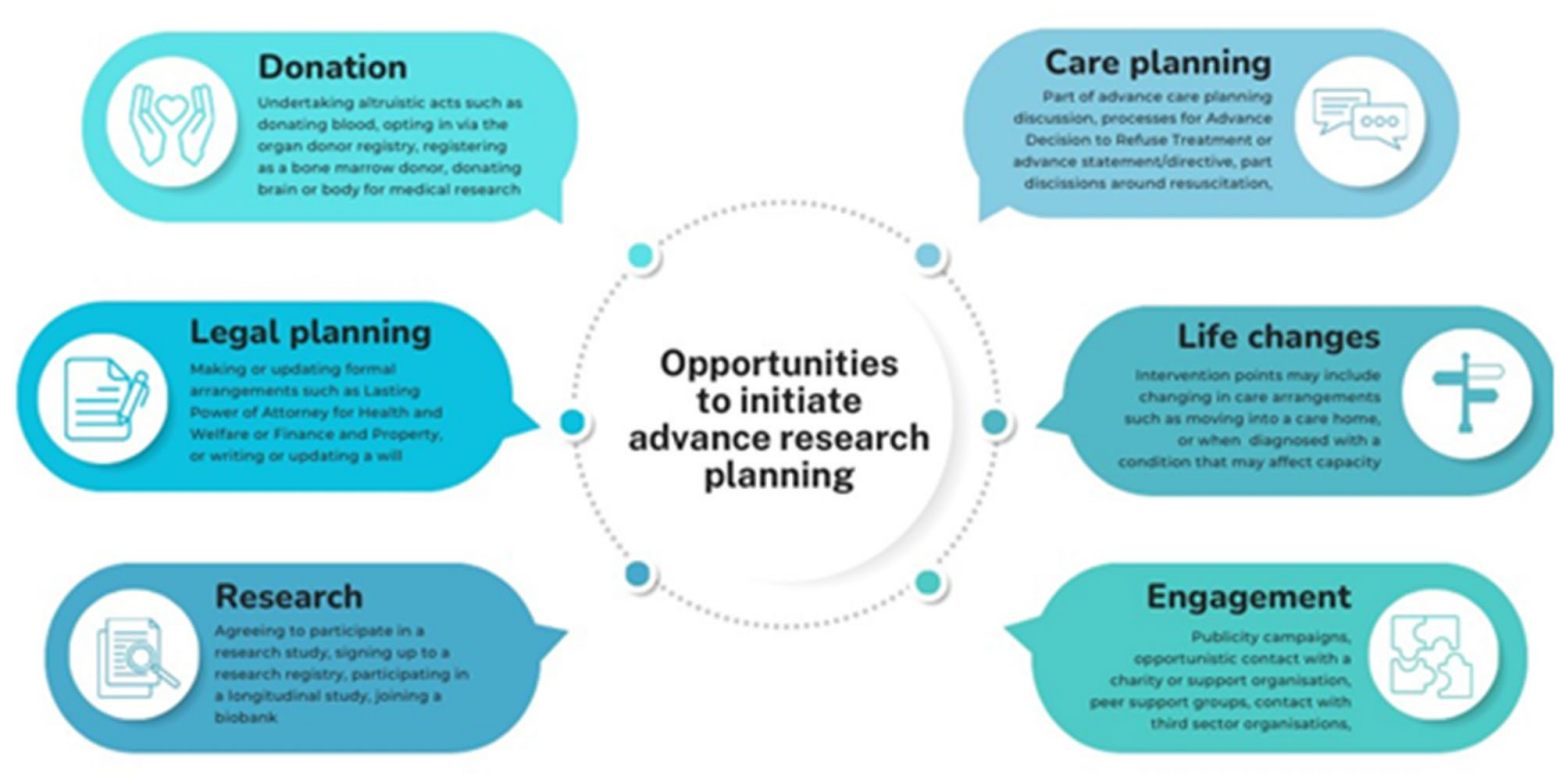
Opportunities for engaging with and initiating advance research planning

Key areas for opportunities to introduce the concept of ARP and signpost to more information are summarised in Fig. 1 below.

Participants suggested that ARP could also be embedded in processes offering care and support for people with a specific condition such as peer support groups or post-diagnostic support or advertised through GP practices or hospitals (e.g. posters in waiting rooms or on websites). Some also described how opportunistic events might also have a role in initiating ARP discussions, such as impromptu conversations with someone who has been involved in research, or arising from high profile media coverage which might raise awareness about the importance of research (e.g. COVID) or the impact of capacity-affecting conditions (e.g. diagnosis of a celebrity).

*‘Because if you get some big megastar who’s got dementia*,* I’m sure there’ll be a lot more people who’ll be involved in the research*,* and then it sort of ebbs and flows. It’s how to nurture that and*,* again*,* you’re just open to almost like the tides really. You can’t control them but maybe harvest them’ [ID 14*,* family member]*.

### Communication and addressing informational and support needs about ARP

Participants stressed the need for all those involved (or likely to be involved) in ARP discussions to be provided with accessible information about research and ARP, which should be available in multi-media formats (e.g online, leaflet, video) and may need to be tailored for different audiences to account for their informational needs and role in the process. Most participants suggested that a coordinated media campaign would be particularly useful to help raise awareness with both the general public and professional audiences.

*‘Maybe like a scheme*,* a campaign like ‘Do you know what your loved ones would want? Have a conversation about it’. And maybe start with like that first and ease people in before writing the form’ [ID 27*,* family member]*.

*‘I think Research Ethics Committees*,* people who already work in research*,* everybody would need more information about the process and what documentation to expect. People with a condition and family members. I think it should all focus on opportunity’ [ID 09*,* member of the public]*.

Participants expressed a range of views about whether engaging in ARP would be a self-directed process or take place within a healthcare consultation or other supported conversation or could be either of these options depending on the context, for example whether the person had been diagnosed with a condition that could affect their capacity in the future. Where ARP discussions would involve health care practitioners or other personnel, participants thought it important that they have the skills, knowledge, and time to enable them to do so effectively.

*‘It would require very good communication skills to introduce it as a topic*,* because it’s part of the bigger context of what’s going to be happening with that person’s condition*,* and illness’ [ID 05*,* researcher]*.

However, participants recognised the challenges of including it in consultations as an additional activity within an already pressurised health and care system. As a result, some participants thought that non-clinical settings may be better placed to provide the time and support that would be needed.

*‘Like all these things*,* it needs to be done with time and explanation and accessibility and that’s where I’d have concerns about who was going to do that conversation and how much time they’d actually have to do it in’ [ID 11*,* researcher]*.

*‘Maybe peer to peer support groups and other community support groups where they’re back every week they can be introduced as a topic and then as a talking point and then*,* people can come back to when they’ve had real proper time for reflection …. it’s a complex idea and a complex thought and I’m not sure that*,* that a clinical setting is necessarily always the best place to go through that’ [ID 12*,* HCP]*.

Given the potential complexities involved, participants thought that ARP discussions might be more effective if they included the family member(s) being nominated as a consultee/representative, where possible. This would ensure that they were aware of the presence of any document detailing the person’s preferences, could use the conversation to help them to contextualise and later interpret those wishes, and could provide continuity in the event of a loss of capacity (e.g. if the person was being cared for in a different setting at that later timepoint).

*‘I think it’s finding engaging ways to make the information really explicit because I suppose the differences are so key*,* and having some kind of way of ensuring whoever is the named representative for that person engages with the process as well’ [ID 06*,* researcher]*.

### Theme 2: a missing part of the puzzle – how preferences expressed through ARP could help inform decisions about participation

Participants often deliberated the acceptability and feasibility of ARP with reference to other types of advance planning activities and other types of research processes, with many highlighting that the absence of mechanisms to include research preferences meant that ARP was a missing ‘piece of the puzzle’. However, for many it was less clear how this new process would align with the legal statuses of the differing arrangements where some are legally binding and others are not, and how it could be integrated into processes for research involving adults lacking capacity where currently an alternative decision maker is involved.

The following three sub-themes explore issues around how binding the preferences expressed through ARP would be considered, how they could inform decisions about participation, and the relative weight that may need to be given to the preferences.

### Outputs of ARP are general sentiments rather than specific decisions

Participants expressed a range of views about how binding they thought preferences expressed through ARP would be. These views were often shaped by whether they were primarily thinking about treatment related research such as clinical trials, or less overtly invasive research such as observational studies, and also how generic or specific those preferences are and how recently they had been expressed.

*‘Are you then looking at the more general sentiments rather than specific decisions. It’s more a general sense of what I would prefer rather than it being legally binding or having that much weight - it’s more of an advisory document that says this is what I would like’ [ID 07*,* researcher]*.

Some participants thought that sentiments expressing openness to research could not be considered ‘decisions’ as it would not be possible to foresee the potential range of circumstances that might apply. This placed the emphasis on people being supported to discuss their general preferences through ARP rather than making specific decisions.

*‘It’s not about making the decision*,* it’s more prompting the discussions’ [ID 23*,* member of the public]*.

*‘If it was a generic document for any research*,* for the future*,* for that person*,* that becomes a little bit more difficult*,* obviously. But if there were guidelines in that document it might make it easier for people to sign it.’ [ID 21*,* family member]*.

However, some HCPs saw greater utility in more specific documented preferences that could be said to constitute informed consent for a study. This was thought to be particularly valuable in circumstances where consent requirements were perceived as creating a barrier to inclusion, for example in emergency situations such as stroke trials, and for observational studies involving routinely collected data.

*‘I think that’s probably a situation where advance consenting would probably be a lot more helpful*,* from a research perspective anyway’ [ID 10*,* HCP]*.

However, other HCPs felt that the range of potential trials, the evolution of clinical trial design, and the speed of developing new interventions meant that it would be difficult to specify what future types of studies and interventions a person would be willing to participate in and receive. This might be particularly relevant if the aims or outcomes of the study might not be what matters most to the person at that point in time. This was particularly stark for those involved in research into conditions where the precipitating event was entirely unexpected, and where clinical outcomes were particularly uncertain, such as in prolonged disorders of consciousness. Participants described the unlikeliness of being able to capture this nuance in ARP.

*‘The treatment turns out not to be very effective so you’ve still gone through the procedure*,* and yet your outcome is still poor and you’ve only consented on the proviso that*,* or you’ve only put in your advanced plan*,* this would be a quality of life enhancing treatment and actually all it’s done potentially is preserved your life but with poor quality of life’ [ID 07*,* researcher]*.

Some participants suggested that preferences expressed through ARP might not be binary in nature, so that rather than establishing either generic or specific preferences, a layered or tiered approach might be preferable. In this situation, people would be able to express their general preferences about the opportunity to be involved in research, with the ability to specify more detailed preferences should they wish, or if they are in a position to do so. This was thought to better align with other types of advance decisions such as organ donation, recognising that these processes encounter similar challenges.

*‘I think maybe stepped kind of consent would be a better idea or stepped permissions because not everybody wants to go for everything. I mean it’s the same with emergency [care] planning*,* like there are some things that people just never want*,* the same with [organ] donation isn’t it’ [ID 11*,* researcher]*.

### Weighing of ARP preferences when decisions are being made

When it comes to how ARP preferences could be used in participation decisions, most participants thought that preferences expressed through ARP would supplement the current process of consulting someone else on the person’s behalf, rather than be considered as a binding decision that ‘bypasses’ the need for an alternative decision maker.

*‘I hope that it would kind of be a parallel process that would eventually inform the consultee process*,* rather than something that would replace it’ [ID 07*,* researcher]*.

Under this view, decisions about participating in a study would be made by someone caring for the person based on their preferences expressed though ARP, but also informed by their current circumstances and involving the person themselves in the decision as much as possible. Honoring the person’s preferences about research, and helping them to fulfil their wishes, was considered to be part of the caring role.

*‘The carer knows the person the best. Nine times out of ten [they] would have been there when they signed the form*,* the carer should have their best interests at heart*,* the carer would have lived with them probably*,* the carer would know what they want …. The person has indicated during their good days that they want to take part in research*,* the carer will help them honour that. The researcher should just talk to the carer and the person*,* because they may have lucid moments*,* or good days or bad days’ [ID 13*,* person living with a condition that may affect capacity]*.

However, one participant cautioned that, whilst the family member’s view about their current situation is important, the person’s previously expressed preferences may need to be given a greater weighting.

*‘Maybe you need provision for somebody close to actually give an opinion of what they think the person might feel*,* but you’d have to have a way of not giving that same weight as the person themselves’ [ID 22*,* member of the public]*.

Whilst the involvement of an alternative decision maker was seen as a safety check by some, other participants felt that where the person was adequately informed (and sufficiently specific) when expressing their views through ARP, their preferences should not be overridden by a family member.

*‘No*,* I think it should be legally binding. That’s how I would rationalise it*,* in essence*,* that thesearethe wishes of this person. If I’m giving unconditional support to the research and signing a document*,* that is my wish’ [ID 17*,* person living with a condition that may affect capacity]*.

*‘If you express a preference to take part in certain research and then your consultee comes along and says ‘no’*,* they’re undermining your wishes but at the same time having that secondary check when the context is known feels like a relief to me…’ [ID 11*,* researcher]*.

### Opting into research versus opting out

There was also a view from some participants that a person’s wish to not be included in research could be viewed as carrying more weight than a wish to be included. Although some participants thought that even when a wish to be included has been expressed, it may need to be ‘double-checked’ by another person who would be able to reduce the level of participation if they felt it wasn’t appropriate given their current condition.

*‘If they’ve said that they don’t want to do something*,* that’s probably considered a binding contract. But then the agreement to opt in …. you should just always seek consent from that person’s power of attorney or whatever*,* because that person’s wishes and feelings may well have changed over time and someone else is better informed to say’ [ID 06*,* researcher]*.

*‘Unless there was anything you want to take off*,* for instance if you no longer thought it appropriate for them to take part in scans because their fear of going to scans would now be too great*,* then I could see it working that way. So*,* you’d say no to some things but definitely not the other way*,* saying yes to something that they had originally said they wouldn’t want to take part in’ [ID 24*,* family member]*.

This contributory or supplementary role of ARP, and its value in emergency situations where participation decisions need to be made quickly and by clinicians who are not familiar with the patient, was recognised by some participants. Having access to previously expressed preferences about research though ARP was thought to be particularly useful in circumstances where a family member was not immediately available to provide their opinion about whether the person should be involved in the study or did not feel able to make a decision.

*‘I see it totally working for a family member who’s a consultee*,* because it’s basically the person’s wishes laid out*,* so they would have no reason to question them… but yeah*,* I think it would definitely remove that kind of lack of confidence that clinicians have in certain contexts*,* including emergency medicine … everybody’s so cautious*,* then if you have this group who have something written down*,* everyone will breathe a sigh of relief’ [ID 11*,* researcher]*.

One participant expressed the view that ARP could play an important role in helping to overcome barriers caused by gatekeeping behaviours and paternalistic views about studies involving people with impaired capacity to consent. This might include families, but also health care professionals and research ethics committees (RECs).

*‘They can make certain ethics committees feel quite uncomfortable*,* and maybe put things in place which actually make it even more challenging to recruit. It’s protection*,* it’s this idea that people are vulnerable and that having them involved in research is burdensome*,* but it’s not. Clinician gatekeeping is a real issue in palliative care research’ [ID 05*,* researcher]*.

### Theme 3: finding the ‘sweet spot’ – optimising the timing of ARP

#### Need to align motivation and opportunity

One of the challenges identified by participants was how to align the opportunity for people to express their preferences about future research with their motivation to do so. Many participants recognised the challenges of people being motivated to undertake any form of advance planning, despite this being widely advocated.

*‘One of the big challenges with advance care planning is that culturally we don’t think a lot these things are going to happen to us*,* and it’s this idea that you’re tempting fate by having these discussions’ [ID 05*,* researcher]*.

Participants also noted that taking part in research isn’t on most people’s radar currently, let alone thinking about taking part in research at some distant time point in the future should they lose capacity to express their own views. Enabling research to seem more relevant to people was described as being the ‘first hurdle’ to overcome, given the general public’s lack of knowledge or exposure to research.

*‘People just don’t think about being in research*,* it doesn’t feel like something that you do as a normal person*,* maybe it feels like something that old people do’ [ID 08*,* researcher]*.

Participants suggested that highlighting the positive benefits of participating in research may help to encourage people to engage with ARP, particularly those who are living with capacity affecting conditions and who may wish to help future patients with similar conditions.

*‘I think that’s what people want to do*,* don’t they*,* it’s that kind of legacy of having lived with something*,* you want to make it then better for other people to not have to go through what you’ve lived through.’ [ID 12*,* HCP]*.

Alongside research needing to seem relevant to people, participants described how the circumstances under which they might not be able to provide their own consent to take part would also need to seem relevant in order for people to engage with ARP. As one participant noted, ‘*it’s not their problem until it’s their problem’ [ID 03*,* HCP].* However, they also noted the difficult nature of these conversations and the challenge of getting the timing right.

*‘I think people should be encouraged to think about this*,* but people are so scared of their own mortality and their own death. If you ask people too early*,* they’re just going to be like ‘what are you talking about?’ like*,* ‘I’m never going to die*,* I’m never going to get dementia’*,* so it has to be at the sweet spot’ [ID 08*,* researcher]*.

#### Impact of delaying opportunities to engage with ARP

Participants with experience of caring for people with neurodegenerative conditions such as dementia also highlighted the need to balance this with not leaving future planning discussions until a point in time where the person was not able to engage fully in the conversation.

*‘Although they may be distressed*,* distraught*,* or whatever because of a life-threatening illness they would be capable of making a decision*,* have the mental capacity I suppose … but down the line you need to have already made the decision*,* otherwise it’s too late’ [ID 26*,* member of the public]*.

However, opportunities for timely discussions could be impacted by wider systemic issues of people experiencing delays in receiving a diagnosis of a capacity-affecting condition such as dementia, and a lack of healthcare support even once diagnosed. This was a view commonly shared by people with lived experience from across the UK, although it was not raised by HCPs.

*‘You really are left alone just to do your own thing. So*,* they don’t go to the doctors unless they’re ill. Getting to the memory clinic is*,* you’re lucky*,* you’re on a waiting list*,* you get one visit and that’s it’ [ID 24*,* family member]*.

*‘He attended the memory clinic …. and he has an appointment like once a year …. and anything can happen within that year’ [ID 16*,* family member]*.

### Theme 4: more than a piece of paper – finding the best modes for recording and documenting preferences

#### Best format to facilitate expression of wishes about research

There were mixed views from participants about the best way to record ARP conversations, and the preferences that were expressed. This often focused on whether a template ‘advance research directive’ document would be helpful in facilitating people to express their wishes, how detailed it should be with respect to specific conditions or types of research, and how it might align with other relevant documents that people might already complete and need to retain.

*‘It gives you something physical to keep with your professional documents*,* you know*,* important papers that should somebody need to be checking through your information*,* at any point*,* just as a reminder to them to use that as well’ [ID 20*,* member of the public]*.

Participants often drew parallels with other processes for documenting advance decisions, including those recording altruistic-based wishes such as organ donation, and those relating to their future care or treatment. Some participants suggested that mirroring the format of other advance directive documents such as ReSPECT forms (Recommended Summary Plan for Emergency Care and Treatment) [40] would be helpful, for example containing a list of options to select from rather than having only open ended text boxes. This similarity in format was thought to help with completing the document and be helpful when it is later used to inform a participation decision.

*‘If you had a list of bullet points on the form that said ‘I’m happy to take part in the following studies*,* observational*,* pharmaceutical*,* device*,* and so on’ then it …. would be easier rather than it being a block of text … and makes it less ambiguous’ [ID 10*,* researcher].*

*‘I think for it to be of best benefit it would need to be mostly generic*,* mostly a tick box*,* that takes away ambiguity*,* it’s easier for people to understand*,* and less of the free text. I think that would protect both sides better and a broader range of people’ [ID 21*,* family member]*.

However, other participants thought a template with various options to select could seem overly burdensome and deter people from considering research all together. Some thought it would be too restrictive and preferred to have the ability to personalise their response using free text or use audio or video recordings which they thought would allow them to better express their own voice in the process.

*‘I just feel that detailed questions may put some people off taking part altogether because they go into too much detail and they start to sound really scary …. whereas some research is not invasive in any way.’ [ID 23*,* member of the public]*.

*‘I wonder if there is a way for me to be able to write a paragraph on my thoughts and opinions*,* in my tongue*,* my words*,* and my vernacular*,* that is making it actually reflective of what I want*,* instead of just legalese tick boxes that*,* isn’t reflective of what I want. Or it is reflective of what I want*,* but it doesn’t sound like what I want’ [ID 01*,* HCP]*.

One participant suggested that another briefer format such as a wallet-sized card to carry would be more effective, particularly in emergency situations, which would state that if the person lost capacity they would still like to take part in medical research.

#### Digital versus paper options – challenges for recording, storing and accessing ARP documents

Linked to this, there were also a range of views about how advance research directive documents should be completed and stored in order for them to be accessible to families, clinical/research teams and to the person themselves. Both paper and digital formats would be affected by issues around access to personal documents once a person had lost capacity affected, but in differing ways. For example, digital formats were considered more secure but couldn’t be accessed without knowing the person’s passwords and might be affected by technological advances whereas paper documents might be more easily accessed but could be lost or become untraceable over time.

*‘We don’t have any documentation because before she came to live with us*,* she was posting stuff off all over the place and getting rid of things. If all of her identification documents were online our life would have been much*,* much easier’ [ID 24*,* family member]*.

*‘If we were to lock [away] information like this about someone who has dementia on a digital system*,* it may be really difficult to get that information without Power of Attorney …. And even if you have Power of Attorney it doesn’t do anything for the password’ [ID 23*,* member of the public]*.

The need for digital versions to keep pace with changes to technology and information governance over time was highlighted by some participants. Ensuring that people discussed their research preferences with families, and informed them that they had completed an advance research directive, was thought to be the best way to reduce the impact of these challenges.

*‘Whatever happens online*,* I feel it’s going to get overtaken by legislation and IT developing and everything …. whatever documents that are finally agreed about research*,* [it is important to] stress that you need to talk to your family about this’ [ID 14*,* family member]*.

The difficulties of being able to access up to date ARP documents, particularly in urgent situations when an enrolment decision is time-sensitive, were frequently raised by participants. Some reported that even accessing electronic health records between different healthcare organisations was impossible in some settings. Several participants referred to the difficulties they already encountered with accessing other forms of future planning, such as LPA documents and advance directives, or even being aware that they had been completed. Communication about the existence of an ARP document was seen as key, along with integrating ARP into existing pathways.

*‘The NHS is getting better at IT*,* but actually the LPA*,* unless somebody comes into the hospital waving it you’ve often got no indication that the person has one in place’ [ID 09*,* member of the public]*.

*‘It’s the same as the ReSPECT form*,* does anybody know you’ve got it*,* is anybody going to look at it. Because if you get a new form as part of a process it has to be integrated into a kind of treatment pathway’ [ID 11*,* researcher]*.

### Theme 5: keeping the door open to future opportunities opportunity – minimising the risk of unintended consequences

#### Impact of uninformed and misinformed decisions about research

Whilst there were high levels of support for ARP, there was recognition that introducing ARP is not without unintended consequences, particularly if people’s preferences about participating in research or not were insufficiently informed. This was linked to the view that levels of awareness and understanding about research are relatively low in the general population.

*‘It would be a travesty if loads of people said they didn’t want to take part in research*,* especially if that was because they didn’t understand the nature of research*,* like*,* what kind of research they were opting out from versus opting into’ [ID 08*,* researcher]*.

One participant described an example where patients at an outpatient clinic had misunderstood the invitation to express their general interest in taking part in research when asked via an electronic screen as part of routine booking in procedures. Patients often clicked different ‘yes’ or ‘no’ responses at each clinic appointment. It demonstrated the difficulties around interpreting binary ‘yes or no’ answers, including where there were inconsistencies in their responses when being asked the same question on multiple occasions. It also highlighted the importance of having contextualised discussions about research rather than just replying on one-way electronic communication.

Some participants drew parallels with existing ‘opt out’ arrangements such as organ donation, and highlighted the negative consequences of any shift towards the view that researchers could only include people who had ‘opted in’ through prospectively expressing a wish to be included in research. Participants were particularly concerned about the impact this would have in terms of exacerbating inequalities in access to research that are experienced by some groups, and thus further reducing inclusivity and diversity in research.

*‘Potentially you’re missing out on huge swathes of people that would not have signed something for whatever reason. Just because people that tend to sign up or are in positions to make advanced care plans*,* might just have less chaotic lifestyles. You know*,* it’s all that kind of the social and economic drivers and determinants of health generally’ [ID 06*,* researcher]*.

There were concerns about how relevant the wishes expressed in an advance research directive could be considered if a long period of time had elapsed since its completion. Particularly as there would, by definition, have been changes to the person’s health status during that time which resulted in a loss of capacity.

*‘I think that’ll be very challenging …. if it’s put in a drawer for ten years and things have changed massively since then how much weight are we*,* as a care team*,* as the researchers*,* going to put in that document when we’re representing the person as they are now*,* ten years later’ [ID 07*,* researcher]*.

Some participants were concerned that the passing of time might also affect people’s recall of discussions about ARP, or even that any discussions had taken place. More fundamentally, ARP would rely on the attitudes towards research held by those responsible for making a decision about research and whether they were trying to make an authentic decision based on the person’s wishes or based it on their own personal views about research.

*‘Those people who are in a place to be more open minded about research participation would be supported by that conversation having had occurred*,* because they want to be part of it and [are] looking for reasons to do so*,* to make that more authentic decision. But then there is a really large group for whom*,* particularly in the early stages*,* even if that conversation had occurred*,* it probably wouldn’t be available in their working memory. Because you might approach those same family six or twelve months later’ [ID 03*,* HCP]*.

### Theme 6: navigating with a compass - principles underpinning ARP to ensure safeguarding and help address inequalities

#### Fundamental role of trust in ARP

Trust was thought to play a key role in ARP, as it does more fundamentally in research. Participants described the relationship between the trust people might put in ARP and the process for engaging with it, and the trustworthiness of the source of information about ARP. This may depend on their level of trust in research itself, and how this might vary between different types of research. Participants described how information about ARP that was received through existing trust relationships, such as with a healthcare provider, might help foster trust in ARP.

*‘There would be quite a lot of people maybe who don’t want to resort to something online*,* because they’ll not be sure if they can trust it or not. Whereas*,* maybe information that they’ve acquired at their GP surgery or in the hospital*,* they will have more faith in that’ [ID 20*,* member of the public]*.

However, given the ‘vulnerability’ of this group, there are implications for how organisations involved in ARP might be perceived. Participants thought that ARP should be introduced sensitively, particularly where people have been diagnosed with a capacity-affecting condition, otherwise it risked eroding trust in these existing relationships.

*‘I’d worry that if we were forcing stuff on people*,* whether that would worsen trust in healthcare … if we’re saying*,* well*,* no*,* we’re going to enroll them*,* because they’ve signed a piece of paper twenty years ago that said that they would love to take part in research’ [ID 01*,* HCP]*.

Some participants highlighted that it is important that ARP takes account of particular concerns around trust associated with certain types of research or research activities, or where levels of trust and views about trustworthiness may differ between communities and individuals depending on cultural, historic, and other contextual factors.

*‘There’s some careful wording where it’s human tissue … or there’s religious and cultural reasons why they would be absolutely dead against something like that’ [ID 06*,* researcher]*.

#### Safeguarding and protection of interests

The importance of ensuring there are safeguards against harm or exploitation was highlighted by many participants, although a number thought that having consultees or legal representatives involved as part of the process already fulfilled this requirement. This included their view that a consultee/legal representative should be able to advise that a participant should not be involved in a study or should be withdrawn from a study if they are distressed or experiencing harm. This balance between the need to protect them from harm as a result of a particular study and the requirement to honour their prior wish to be included in research more generally was described by one family member as the ‘ethical cut off’ point. This limiting of harm or distress was seen by participants as being part of the role of enacting the person’s wishes as it is unlikely that they would have agreed to participate if it involved such harm.

*‘I think I would want a safeguard in place where someone could step in and say actually things have changed to such an extent this would cause distress and actually if this person had capacity*,* they would acknowledge that themselves and so we don’t think it would be appropriate for them to take part’ [ID 02*,* researcher]*.

*‘So*,* I think if it comes to a point where somebody’s health is rapidly declining*,* because of the research*,* that’s when there should be that family who says*,* “no*,* they’re not coming anymore”.’ [ID 21*,* family member]*.

Some participants thought that this situation would need to be articulated as part of the person’s preferences about being involved in research, although one recognised that putting caveats around what was acceptable in terms of distress would make the ARP process difficult, and probably end up making it almost impossible to operationalise. Others wondered if a third party organisation could provide an independent review in situations where there were disagreements or concerns, although it was suggested that their role would be supportive or mediation rather than having decision-making authority.

*‘I wonder if there was a process for maybe a voluntary service …. where independent advisors go in and review the situations and review what the family are saying or what doctors are saying and make decisions. Could they overrule a next of kin? Probably not. So*,* like some independent service*,* that offered support and guidance*,* in these kind of tense situations*,* would probably be really good.’ [ID 01*,* HCP]*.

Other participants expressed the view that the arrangements in place around the person’s care and treatment will already be playing a role in safeguarding, as someone who lacks capacity to consent will be requiring care and support outside of a research context.

*‘There would already be health decisions that’d have to be made outside of just this to have flagged up that this person was particularly vulnerable …. I’d find it difficult to imagine a position in which they wouldn’t already be known to the system as it were.’ [ID 06*,* researcher]*.

There was also the view that, unlike other areas such as finance where people may have motives to exploit ‘vulnerable’ people, research participation is unlikely to create the same ulterior motives.

*‘There’s less immediate material gain in research*,* so I can’t really imagine somebody being able to benefit directly from having control in that way’ [ID 06*,* researcher]*.

Participants also highlighted the well-established safeguards that are in place around research conduct, including the requirements for ethical review and existing legal protections in terms of data protection and confidentiality, and the additional safeguards that govern research involving adults who lack capacity to consent. They suggested that communication aimed at raising awareness about ARP should include highlighting that there are existing safeguards in place and that its purpose is not to bypass or subvert these important protections.

#### Not perfect, but better that the status quo

Despite the complex issues raised, introducing ARP was generally seen as an improvement on the current imperfect situation. It was seen as adding what one participant described as an ‘extra layer of security’ that would make the process more ethically robust for all those involved in comparison with the current arrangements.

*‘I think at the moment using proxy consent is often used as the best way forward*,* but I think it’s imperfect. I would see something like advanced research planning as an improvement on the current status quo. If our research relates to vulnerable groups who may not be able to give consent*,* we’re trying very hard to think how we can best respect their dignity by including them earlier on in the conversation*,* but then have a safeguard whereby their consent can be overridden in very particular circumstances. That would strike the right balance*,* I think’ [ID 02*,* researcher]*.

Importantly, a number of participants stressed the risk of potentially reinforcing existing inequalities in research that would need to be addressed during implementation. These may arise through the routes to implementation that may not reach people from under-served groups, and around issues relating to the accessibility of ARP processes themselves.

*‘There’s a huge selection about populations being under-represented by self-selecting to take part in these things and actually they’re the people we get to too late already because their needs are so very complex and we need more research to help them*,* to get better health outcomes than they currently do’ [ID 12*,* HCP]*.

*‘Anything that involves digital literacy*,* online engagement*,* you’re going to disadvantage certain people. If it’s only located in primary care*,* you’re going to disadvantage populations like the gypsy and Roma that move a lot and disengage with services. If it’s only done in English. I think most standard strategies*,* through standard pathways are going to exacerbate the historically excluded groups’ [ID 11*,* researcher]*.

There were particular concerns around the impact on inequalities of any ‘ethics creep’, for example if it became a *requirement* that people with impaired capacity to consent could only be included in research if they had an advance research directive in place.

*‘My underlying catastrophising around some of these things is that if we set up an advanced research plan or advance research directive*,* as a requirement for participation*,* we are going to filter out anyone but the very*,* very most informed who will undoubtedly be the tertiary educated white*,* well-paid*,* well-represented people in research. It’s going to become naturally exclusive through its attempts to include’ [ID 03*,* HCP]*.

#### Ensuring accessibility of ARP

A number of strategies were suggested by participants to enhance the opportunities for historically under-served populations to be able to engage with ARP. These included ensuring that the format of ARP is accessible. It was also thought important that all communication aimed at raising awareness about ARP uses accessible language to avoid the risk of these groups not understanding the purpose of ARP (and thus make an uninformed decision) or not be able to engage with it.

*‘I think a booklet’s a good idea. I think that’s an accessible resource to all. You could make it in different languages*,* different print for those visually impaired. I think a website*,* also. I think it needs to be very clear about the different types of research that they might be involved in …. to explain all those terms*,* the kind of layman’s terms*,* so they can understand what they may be involved in*,* and then for them to make an informed decision of what they do and don’t want to do going forward’ [ID 04*,* HCP]*.

Participants also stressed the need to ensure that information is accessible for all the groups and individuals likely to be involved in ARP, including those in clinical roles who may themselves be less familiar with research.

*‘You don’t want to assume that much research literacy in clinicians*,* and you don’t know the level of understanding of the consultee either. You can’t assume it’s better or worse than anybody else*,* so I think focusing on less being the key and accessibility is the best way to go’ [ID 11*,* researcher]*.

## Discussion

This study found high levels of support for ARP as a mechanism to enable people to express their preferences about being involved in research should they lose capacity to consent in the future, and for those preferences to inform participation decisions. Participants viewed ARP as having an important role in enabling people to continue to have the opportunity to be involved in research should they wish, or to be able to state if they would not wish to be involved, which they are currently unable to formally express in a readily recognised format. Participants also valued the ability to identify a trusted person who in their view would be best able to represent their wishes, which is in contrast to the current situation where an alternative decision maker is identified and appointed by others once the person themselves has lost capacity [15, 16]. Researchers had experienced the impact of family members being unable to draw on a previous discussion to help them to make a decision about research participation, and some shared examples where a form of ARP had occurred in practice as has been reported in the literature on palliative care research [32]. Many participants drew parallels with other well established advance planning arrangements that enable people to plan for future changes in capacity and viewed it as an obvious step to extend this to include preferences about research. Whilst not viewed as a ‘perfect solution’, it was generally seen as an improvement on the current situation where people’s research preferences are often unknown [41] and decision makers rely on ‘informal evidence’ [42], which leads to concerns about ‘over-enrolment’ and ‘under-enrolment’ [17]. Previous studies found that, of those completing an advance research directive, the proportion who used it to express a wish not to be included in research were 13% [42] to 15% [17].

However, there were differing levels of support for the spectrum of activities that may come under the umbrella of ARP, and about how determinative the views expressed would be, which depended on the context and the anticipated risks and benefits of participating. In this study, and similarly in the preceding survey [23], participants generally viewed ARP as a process to encourage people to express their values, wishes and preferences – rather than it constituting an advance decision. This was based on their view about the likelihood of having sufficient information upon which a ‘decision’ could be made, as reflected in other studies describing the challenges of ‘predictive ability’ and anticipating future decisions that may particularly affect people with cognitive impairment [43], and their uneasiness about balancing (or prioritising) a person’s previously expressed decision to participate against what is in their current interests. Some participants accepted that there was utility in more specific preferences which could constitute informed consent for a study, for example in time-critical trials where there is no ability to involve alternative decision makers. This is the focus of ongoing studies in Canada that are exploring the acceptability and feasibility of advance consent in stroke trials [44, 45].

Participants generally saw the value of ARP being in the conversations it could initiate between patients and their family member(s) who would act as an alternative decision maker in order to prepare them for making a decision about research, which could then be summarised in a non-binding advance research directive and used to inform participation decisions. As with previous studies, participants generally would prefer a template advance research directive document [46]. Advance research directives were also viewed as being useful in scenarios where people did not have family members who could be involved in ARP conversations, or where they were not able to be consulted due to certain constraints such as during emergency research or visiting restrictions as seen during the COVID-19 pandemic. However, participants recognised the challenges of interpretating those wishes, particularly given the passage of time and events, and that care would be needed to balance the specificity of those preferences with their utility to actually inform participation decisions, as is similarly a consideration in other advance planning arrangements [47].

Some participants supported a layered approach to an advance research directive in which people could express their general preferences about being involved in research and provide more detailed preferences should they wish, which aligns with other types of altruism-based advance planning such as organ donation, whilst recognising that these processes encounter similar challenges about how these wishes are interpreted in practice [48]. Participants also highlighted the need for preferences to be updated as required, and that the format and storage of any advance research directive would need to have the accessibility and functionality to enable this. These findings mirror the evolution of advance care planning (ACP) which has seen a shift away from its origins as *legal documentation* of a narrowly defined list of preferences about various life-sustaining treatments, towards its current conceptualisation as a *process of preparation* for people and their alternative decision makers through encouraging communication that can support future medical decision-making [47]. It also reflects pervious trials of advance research directives that suggested families made decisions based on their discussion with the person rather than the contents of the booklet [17].

The findings reflect the complexity of the ethical and legal provisions for ARP across jurisdictions, and the variations in the weight given to antecedent preferences. For example, in Canada the Tri-Council Policy Statement: Ethical Conduct for Research Involving Humans enables people to create an advance research directive and states that authorised third parties ‘should be *guided by these directives* during the consent process’ (Art 3.11) [49]. In Australia, the position in law differs between states and territories [11], for example in Western Australia a person can make an advance health directive that ‘includes *a decision to consent or refuse consent* to the commencement or continuation of the person’s participation in medical research’. In Germany, prior to inclusion in a non-therapeutic research study, participants who lack capacity *must have previously declared their wish to participate in a research advance directive* [46]. As other authors have noted, these variations are due to the cultural and contextual differences regarding health policy and law in these countries [46], and varies according to the permissible level of harm or burden for participants when balanced against the likely benefits for the person and/or the patient group.

The importance of acknowledging different cultural perspectives about the acceptability of ARP and ensuring that information and resources are accessible and take account of these diverse perspectives, was seen as key to demonstrating the trustworthiness of ARP – and not adversely affecting existing trust relationships. There is likely to be learning from research exploring other forms of advance planning such as Advance Choice Documents/Advance Statements (ACDs/AS) which are intended to give people with severe mental illness influence over their future care [50], but where there may be particular barriers in uptake by Black people due to issues around trust in mental health services despite ACDs/AS being particularly important for these groups given the poorer care and outcomes they experience [51]. It is also important to acknowledge the rights of people with disabilities, including cognitive disabilities, to have the freedom to make one’s own choices [52], with ARP being seen as part of a wider package of support that should be provided to enable people to have choice and control.

Participants were generally reassured by the existing safeguards around research involving adults lacking capacity to consent, such as that their interests must always outweigh science and society and nothing must be done to which they appear to object [8]. Unlike concerns raised elsewhere [25], participants did not express concern about including research preferences in healthcare discussions or integrating ARP into other advance planning activities which could lead to misunderstandings about the aim of research (primarily to generate knowledge for the benefit of others) compared with healthcare (to benefit the person directly) and so risk ‘therapeutic misconception’.

In line with previous studies, there were concerns about the unintended consequences of ARP. This included concerns that an advance research directive could become a requirement prior to including people who lack capacity to consent as is the case in some other jurisdictions for certain types of research [11]. Participants recognised the impact this could have on the ability to conduct research as expressed in previous studies [42], particularly given the low levels of research literacy and awareness in general populations [53], and that it could result in research becoming *less inclusive* as a result. There were also concerns that people might express uninformed preferences or decisions, reflecting concerns in previous studies [42]. Participants highlighted the need for information and resources to support the uptake and use of ARP to be widely available and easily accessible. Participants did consider that clinician involvement is important for ARP involving people who already had a diagnosis of a capacity-affecting condition such as dementia, where there might be particular sensitivities or where clinical questions might arise. However, the likelihood of busy clinicians being available for discussions about research, or having the knowledge to support it, is less clear. There was also support for it to be available as a self-directed process that would maximise the opportunities for people to engage with ARP at a time and way of their choosing. This would align it with other opportunities to express an interest in taking part in research such as national registries like Join Dementia Research (https://www.joindementiaresearch.nihr.ac.uk/) and the NIHR’s flagship initiative ‘Be Part of Research’ (https://bepartofresearch.nihr.ac.uk/).

Issues around implementation, and the challenges that might be encountered, were frequently discussed by participants and reflected those raised in previous studies. This included concerns about low uptake, which in previous studies of advance research directives with different populations found completion rates ranged from 80% of older people in Canada in a dyad with a family member [17], to 11% of people admitted to a US hospital [42], and 16% of people with a family history of Alzheimer’s disease [54]. Even in studies where there was a higher uptake of the advance research directive, it was found not to be effective in increasing proxies’ ‘accuracy’ (their predictive ability) in hypothetical scenarios [17]. However, these previous studies did not appear to use a behaviourally-informed approach to the development and implementation of the advance research directive, nor use approaches to its development and evaluation that recognised ARP as a complex intervention [55] set within wider social, cultural, and ethico-legal systems, and the outcomes used may not reflect the aim of ARP which is arguably to support authentic decision-making rather than improve ‘accuracy’ [56].

Participants in this study described a need to align peoples’ motivation(s) to consider and express their wishes about future research, with the opportunity to do so (see Fig. 1) and having the capability (resources and support). This was seen as essential to ensuring the successful implementation of ARP in the UK. With a global ageing population, the number of people living with multiple long-term conditions is rapidly growing, including conditions associated with cognitive impairment. Aligned with this, research is becoming increasingly complex, including the use of pragmatic trials and those using platform designs and data science approaches, therefore the need for mechanisms to enable ARP is becoming greater. Participants identified a number of strategies to support the implementation of ARP and address inequalities. The key strategies are summarised in Table 2, alongside examples of similar strategies already in action for a range of other related topics.

**Table 2:** Strategies to support the implementation of ARP and address inequalities

Strategy	Description of facilitator	Illustrative quote	Resources required to support implementation	Examples of similar strategies in action
Use of targeted messaging and support for individuals and organisations	Implementation of ARP will rely on introducing it as a concept, and using a stakeholder-informed approach to provide information about why and how to engage that aligns with different stakeholders’ informational and support needs. This will include those likely to be directly involved in ARP discussions, as well as more broadly across care and research ecosystems, and beyond.	*‘You’ve got to have branding*,* a logo*,* a snazzy title*,* you’ve got to tell them why it’s important’ [ID 13*,* person living with a capacity-affecting condition]* *‘I think that whilst it would be nice to have it more generic*,* I think the uptake would be better if it could be more specific. I think sort of targeting it would be better’ [ID 12*,* HCP]*	A unified and co-ordinated approach is needed, including stakeholder mapping, the development of a behaviourally informed suite of information and resources to support ARP (including training), and an integrated communication and marketing strategy. However, organisations that have strong associations with particularly relevant populations could help target and amplify the key messages.	**Advance Care Planning Canada** is an organisation which has coordinated and innovative strategies to support the uptake of advance care planning (ACP) in Canada. This includes a national ACP Day with taglines such as ‘If not you, who?’ There is a suite of resources for health care professionals, lawyers, and members of the public. Link: https://www.advancecareplanning.ca/
Building inclusive community engagement and support to help widen opportunities for engaging with ARP	Communities can play a key role in raising awareness about ARP and supporting opportunities for people to engage with ARP. They can also help to ensure that these opportunities are open to groups who are currently under-served by research and are less likely to have access to ARP opportunities if they are embedded solely on traditional research and care services and infrastructure.	*‘It is working with those community groups within those communities that don’t engage well with health services (or with any organisational services) because they feel excluded*,* or they don’t know they’re there*,* or they don’t know how to navigate it. To get their engagement you really have to reach in and be prepared to take that time to go back and …. it is finding people within those community groups who are willing to advocate for research and advocate for taking that message … and saying ‘this is about you’’ [ID 12*,* HCP]*	Engaging with communities and community organisations will require appropriate support and resources to avoid overburdening them and to minimise the risk of any unintended consequences (e.g. misinformation, impact on trust).	The **PANORAMIC study** is a UK clinical trial testing different antiviral treatments for COVID-19 in the community. Underserved communities, such as ethnic minorities and people with learning disabilities, have been disproportionately affected by the COVID pandemic and so it was important to ensure that everyone has the opportunity join the PANORAMIC study. The study team worked with a diverse range of community groups to provide resources to raise awareness about the study. This included videos of translated information about the study, and accessible versions of the participant information sheets. Link: https://www.panoramictrial.org/community-outreach
Signposting to ARP by relevant organisations	Organisations providing information and support about health, wellbeing or research can also play a valuable role in raising awareness about ARP and supporting opportunities for people to engage with ARP if they wish. These might include condition- or population-specific organisations, those supporting volunteering activities more broadly, or those currently sharing research opportunities. They could provide introductory context-specific information tailored for their audience(s), alongside links to core information and ARP resources which can be accessed by those who are interested.	*‘I think if people are already generally looking at those things*,* then that probably would be a useful place to put a link that then takes them to another website if it is something that they’re interested in’ [ID 12*,* HCP]*	Building on the core suite of information and ARP resources, organisations should be supported to co-produce context-specific information with those who access their services. A feedback loop will also need to be established to ensure that the context-specific information and core ARP resources are harmonised in line with feedback from those accessing them and updated when required.	Many organisations provide information and resources about making a **power of attorney**, including charities that support people with potentially capacity-affecting conditions. This includes Alzheimer’s Society who have a webpage of resources, including videos and audio versions of the information. For people who don’t have access to the internet, or don’t feel able to complete the forms online, Alzheimer’s Society offers a telephone support line. The forms are completed on the person’s behalf by a trained volunteer using an official online tool created by Office of the Public Guardian. Link: https://www.alzheimers.org.uk/get-support/legal-financial/lasting-power-attorney
Integrating ARP with other advance planning and research activities	ARP requires building on an initial foundation of motivation, capability, and opportunities which may arise through other activities around advance planning, legal arrangements, research participation, or more general altruistic or community engagement. Integrating ARP opportunities with these will increase both the reach and accessibility of ARP activities.	*‘There’s an opportunity to kind of create a bundle together*,* the discussions [about organ donation*,* blood donation*,* LPA*,* participating in research*,* etc] and raising awareness’ [ID 23*,* member of the public]*	Integrating ARP with other activities will require a systems-level approach to map the processes and partner organisations involved, and identify opportunities for embedding ARP. It will require cross-sector working to establish these new pathways and the use of theory-informed approaches to explore issues around usability and sustainability.	**Western Australia’s Advance Health Directive (AHD)** which enables people to communicate their preferences about their medical treatment was recently amended to include decisions about the types of medical research people would consent or refuse consent to take part in. It can be completed in paper or electronic formats, and there is an accompanying guidance booklet and short instructional videos to help people to complete it. Once completed, it can be uploaded to a central portal with other health records. Link: https://pch.health.wa.gov.au/en/Healthy-WA/Articles/A_E/Advance-Health-Directives
Create tools to support people to involve their family members in the ARP process	Involving family members in the ARP process may support people to think about research, express their preferences about taking part in future research, revisit and update those preferences if they wish, and facilitate the use of an advance research directive if completed.	*‘Being part of that discussion would certainly help family members to feel able to carry out those wishes*,* as opposed to somebody else finding a piece of paper saying*,* ‘Well you know*,* your Mum said this’ and it’s like ‘We don’t know anything about that*,* where’s that come from’’? [ID 12*,* HCP]* *‘The chances are by having signed the form they probably will know about it’ [ID 10*,* HCP]* *‘It relies on the consultee understanding research and having had a conversation with that person about the boundaries of their preferences’ [ID 11*,* researcher]*	Involving a family member or another trusted person in ARP discussions will help support the person to effectively engage with ARP and ensure that they are aware of the person’s wishes and whether they have completed an advance research directive. This will help prepare the family member to act as the person’s consultee/representative, including highlighting that a document has been completed and providing access where possible. It will also help them to interpret the preferences it contains within the context of the person’s current situation, thereby ensuring that participation decisions align with their wishes. Resources to support ARP will need to include information and guidance for family members.	Although the processes for **organ donation** have changed in recent years across the UK nations, the NHS has a central website of information for members of the public. This includes information about the choices for opting out and gives a clear message that people should talk to their loved ones as they will always be involved. It includes a series of tips for how to have a conversation about organ donation, such as ‘finding a talking point’. Link: https://www.organdonation.nhs.uk/

### Strengths and weaknesses

Building on previous international research exploring ARP processes in different jurisdictions, this study explored the views of public and professional stakeholders from across the UK. The study has a number of strengths, including the sampling approach which included diverse participants with a range of personal and professional perspectives. This allowed us to explore issues relating to different groups of people with capacity-affecting conditions and disabilities, including acute events that may suddenly affect people’s capacity to consent and people with longer-term cognitive impairment. However, selection biases may have occurred, including that participants may have had a more positive view of research than the general population given that they had previously participated in an online survey [23] and which included recruitment via research registries.

We included participants who were living with a capacity-affecting condition (or that may affect their capacity in the future), however we did not include the experiences and views of people who were unable to consent to the study as this would have been challenging given the nature of the questions and the focus of the study was to explore people’s views about ARP at a point in time when the person has capacity. Participants had previously completed an online survey and expressed an interest in being contacted to take part in an interview. Potential participants’ capacity to consent was assumed in accordance with the Mental Capacity Act [15], and there were no concerns raised when contacting participants for an interview and none were excluded due to concerns about their capacity.

Limitations may include the gender imbalance and lack of ethnic diversity in our study and the number of participants from each stakeholder group, given that we sought to include a broad range of perspectives and inequality was a key theme. However, the use of reflexive thematic analysis [38] and concurrent data collection and analysis provided us with a richness of data (defined as the depth, diversity and complexity) that enabled us to answer our research questions and confirm that sufficient data had been collected [39].

This study was intended to explore stakeholder views about the acceptability and feasibility of introducing ARP in the UK and identify barriers and facilitators, therefore the methodology used does not enable ethical analysis of ARP. Further research is needed to determine the moral authority of ARP.

## Conclusions

This study found high levels of support for enabling people to consider and express their preferences about being involved in research should they lose capacity to consent in the future, with many participants viewing this as an important but currently missing step between people’s research preferences and other expressions of future wishes. The value of ARP may lie in preparing a trusted family member to make decisions about research participation in the event that a person loses capacity to communicate their wishes for themselves. Recording those wishes in a (non-binding) template document such as an advance research directive may act as a reminder about the discussion at a later point in time, and provide an insight into the person’s values, wishes and preferences where that may be the only available ‘evidence’ of their research preferences. Alternative decision makers can then use these preferences to inform their decision, akin to the role of an advance statement in England and Wales [10]. More precise preferences relating to a specific study or context may be acceptable under particular circumstances, and previous studies have suggested that an advance research directive could include the degree of leeway that the person grants to their alternative decision maker [17], although that wasn’t raised in this study. However, in all cases, people should have the ability to revisit and amend their preferences, and their interests must always be protected.

Implementing ARP in the UK will require changes to policy and practice to better address the current gaps between the ability of people to express their values, wishes, and preferences about their future care, and regarding future research participation. This current lacuna between people’s ability to express their preferences about research compared with all other matters relating to their health and care has no sound basis, and the rising number of people affected by cognitive impairment brings a sense of urgency to this neglected area of research in the UK.

Future research should focus on using theoretically informed approaches to developing interventions to support ARP, which take account of the UK’s societal, cultural, and ethico-legal context in which such interventions will be embedded. The use of implementation science and accessible design, underpinned by ethical principles such as trust, will be key to ensuring that this promising contribution to supporting people’s autonomy is actually translated into practice. This will help to ensure that research is accessible to all and that in the future, participation decisions are made more closely in line with people’s wishes and preferences.

### Electronic supplementary material

Below is the link to the electronic supplementary material.

Supplementary Material 1

## Data Availability

The dataset generated and used in this study is available through submission of a data request to the Centre for Trials Research at https://www.cardiff.ac.uk/centre-for-trials-research/about-us/data-requests.
