# Supplementary material for: ‘It’s not making a decision, it’s prompting the discussions’: a qualitative study exploring stakeholders’ views on the acceptability and feasibility of advance research planning (CONSULT-ADVANCE)
Source: BMC Med Ethics. 2024 Jul 23;25:80. doi: 10.1186/s12910-024-01081-5 (PMC11265470; doi:10.1186/s12910-024-01081-5)
Supplement: Supplementary file 1 — Supplementary Material 1 [file 12910_2024_1081_MOESM1_ESM.docx]

**CONSULT-ADVANCE Study Interview Topic Guide**

**1. Introduction**

- Introductions and thank the participant for taking part.
- You have received the information sheet. The main aim of this interview is to get your views about advance research planning – a process where people can express their wishes about being involved in research studies in the future - and what might help or not help this process in practice.
- What we talk about today will be kept confidential. We might use some quotes from what you’ve told in presentations and publications, but your name (and anyone else’s name) will not be used.
- If there are any questions you don’t want to answer, or if you would like to stop the conversation or recording at any time, please let me know.
- I may also make some notes during the interview to add more information to what is being said.
- Do you have any questions?
- If you don’t have any other questions, I’ll start by reading the statements on the consent form and asking you to verbally confirm that you agree with each one.
- Start audio-recording
- Obtain consent
- Stop recording and start new audio-recording

**2. Experience and background knowledge**

1. Please could you start by telling me a bit about your background and why you are interested in talking about advance research planning?
2. How long you have been in this role/situation?
3. What (if any) is your experience of being involved research?

*Prompt for professionals: Are you involved in designing, conducting, or reviewing research studies? Do they involve populations who may have impaired capacity to consent?*

*Prompt for public: Have you, aside from this interview study, ever participated in a research study? If yes: What was your main motivation to participate, what was your experience of taking part? How did you get involved in these studies? What kind of studies are they? How did they come about?*

1. What (if any) is your experience of being involved in advance planning – for example for care or treatment decisions, or Power of Attorney arrangements?
2. Have you already heard about Advance Research Planning? In what context?

**3. Views about Advance Research Planning**

Informed consent is an important requirement for participation in research (*e.g* *as you have just experienced for yourself*). Some people, for example those living with more advanced dementia, are often not able to provide their own informed consent. In this situation, someone else – usually a family member or close friend – is asked to help decide whether they should take part. Family members often don’t know whether the person would want to take part or not as it is not something families usually discuss. Some countries such as America, Australia and Germany have introduced Advance Research Planning, particularly for dementia research, because consent decisions made solely by other people such as families or friends are not always seen as the best approach.

1. What do you think of the idea of Advance Research Planning? Do you think it will help or hinder research in conditions such as dementia, and if so, for what reasons?
2. Do you think there are situations when might it not be appropriate?

*Prompts: Will it help family members who are involved in making decisions? Will it help to ensure that decisions about research are based on the person’s preferences (rather than the family member’s)? Are there some groups of people or some types of research where it should not be used?*

**4. Views about implementation of Advance Research Planning**

1. How detailed could and should Advance Research Plan be to make it useful in practice (e.g include nominating who they want to be involved in future decisions, record the person’s general willingness or unwillingness to be involved in research or more specific)?
2. Should it be considered advisory, or should it be legally binding – or something else?
3. Is there any particular information that the public would need to empower them to undertake Advance Research Planning? Where, how and who should provide it?
4. When would be the best time to ask people to consider Advance Research Planning and who should be involved?
5. Do you see any particular ethical, legal, or practical challenges to implementing Advance Research Planning? Might there be any unintended consequences?

*Prompts: What information should be included, and in what format? Should there be one ‘template’ or condition/situation specific versions? How will people know that this is an option – where could the find information about it and what do they need to know? Should it be considered at the time of being diagnosed with a particular condition? Should anyone over a certain age be encouraged to do so, or when undertaking other advance planning activities such as writing a will or PoA arrangements? Should it involve their doctor or solicitor – or someone else? Should it be revisited (and if so how often/when)? Where and how should it be held and shared? Might there be any costs or inconvenience involved? What safeguards might need to be in place (e.g if signs of distress)?*

**5. End of interview**

- We’ve covered all of the questions I wanted to talk about today, is there anything we haven’t mentioned that you would like to say?
- Thank you very much for taking the time to talk to me today
- Stop audio-recording

**6. Debrief**

- Thank participant for taking part in the interview.
- Reminder that we will keep all the information confidential.
- Do you have any further questions?
- I will be in touch once all the information has been analysed to send you a summary
- You have my contact details - if you would like to get in touch in the meantime, feel free to contact me
- Offer vouchers
